# Supplementary material for: The viscoelastic properties of Nicotiana tabacum BY-2 suspension cell lines adapted to high osmolarity
Source: BMC Plant Biol. 2025 Feb 25;25:255. doi: 10.1186/s12870-025-06232-3 (PMC11852555; doi:10.1186/s12870-025-06232-3)
Supplement: Supplementary file 1 — Supplementary Material 1 [file 12870_2025_6232_MOESM1_ESM.docx]

# Supplement

1. **The relative to medium BDP-rotor lifetime**Δ presented in percents enabled us to show cytoplasmic crowding in context of simultaneously measured media properties. PEG-BDP was shown to efficiently work in broad range of viscosities within tested water-glycerol mixtures (Michels et al., 2020).

$$\mathrm{Delta}(\Delta)=\frac{cytoplasm mean lifetime}{medium mean lifetime}$$

PEG-BDP lifetime of BY-2 cell lines adapted to intermediate mannitol and NaCl concentrations

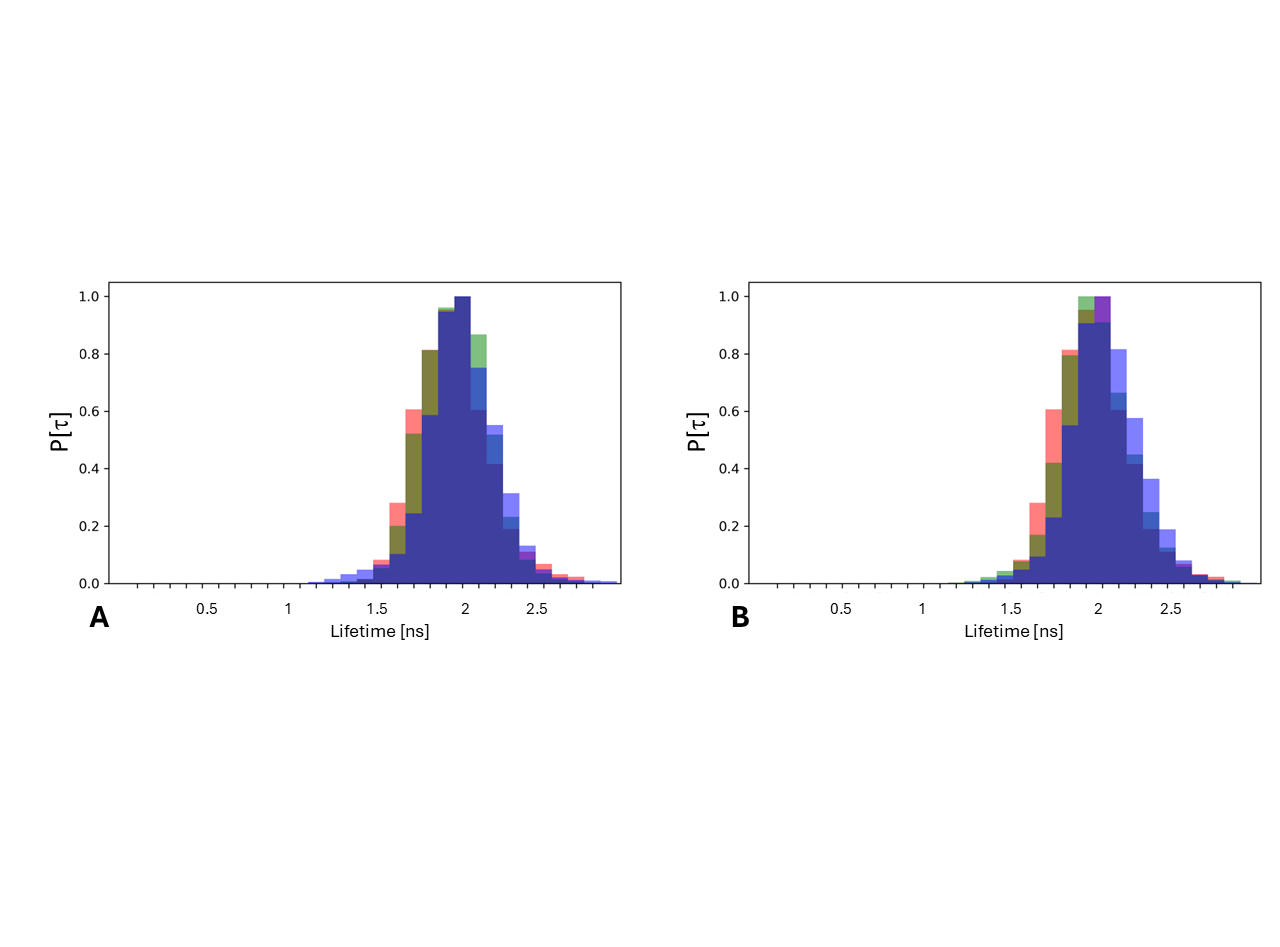


Fig. 1. A) PEG-BDP lifetime histograms of BY-2 adapted to mannitol in respective concentrations: 450 mM (red), 350 mM (green), 150 mM (blue). B) BY-2 adapted to NaCl in respective concentrations: 220 mM (red), 110 mM (green), 150 mM (blue)..

1. The time lapse lifetime imaging of mannitol and NaCl stressed BY-2:Control cells stained with PEG-BDP. The same cells analysed in 2-35 min. time.
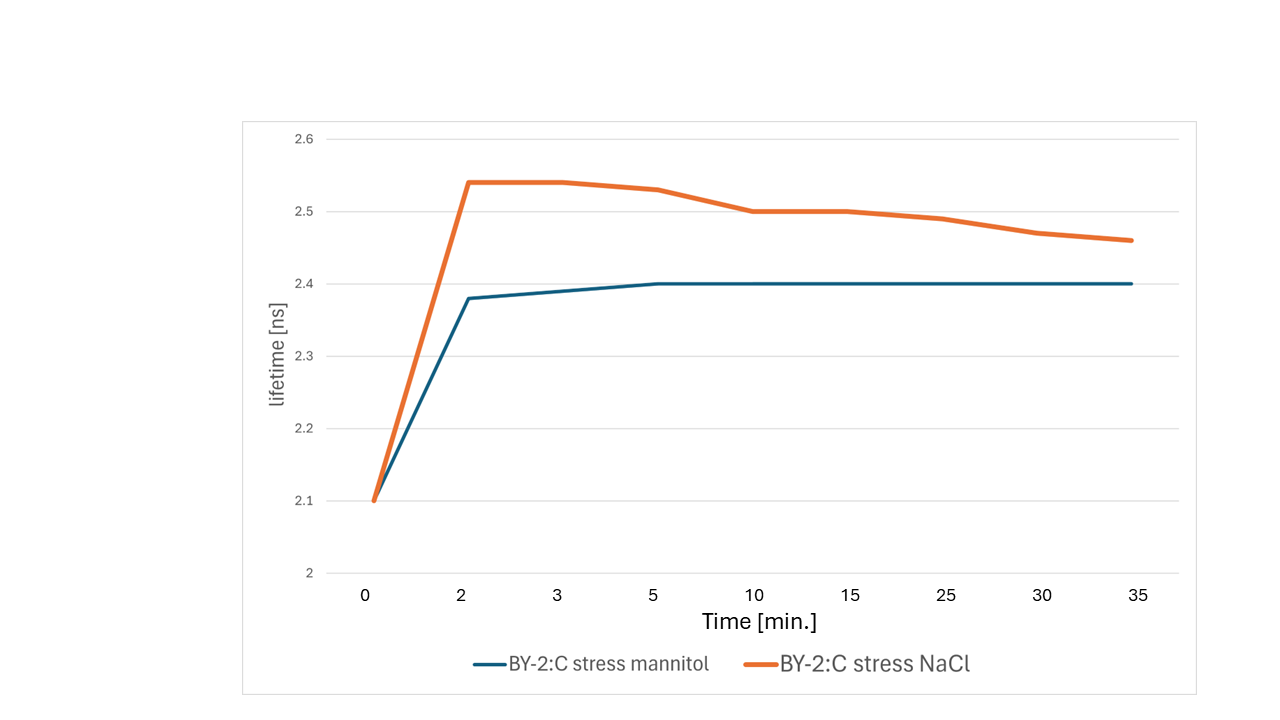


Fig. 2 . Time course of PEG-BDP mean lifetime of the same BY-2:Control cells after NaCl and mannitol stress. Lifetime StDev in range ±0.12-0.17 ns for NaCl stress; ±0.13-0.21 ns for mannitol stress**.** Lifetime measured 2, 3, 5, 10, 15, 20, 30, 35 min. after NaCl stress had started

4) The time lapse lifetime imaging of NaCl stressed BY-2 cells stained with N^+^-BDP. The same cells analysed in 2-35 min. time.


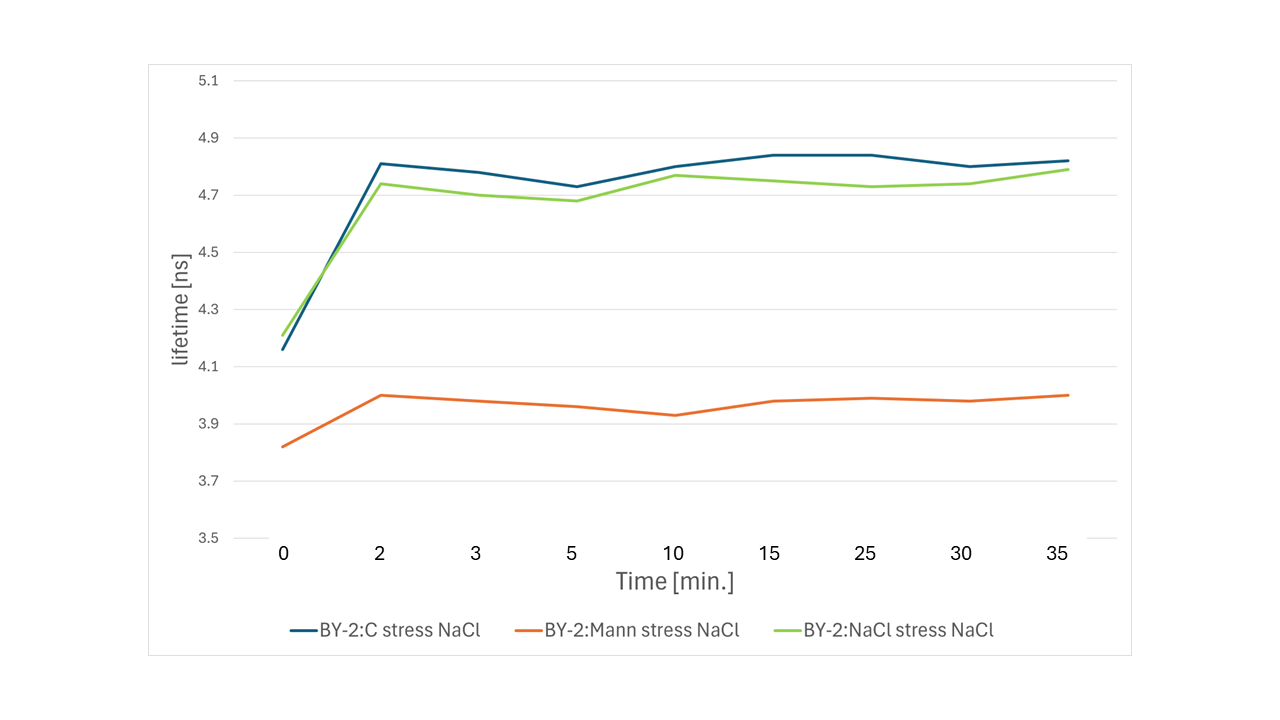


Fig. 3. Time course of N+-BDP mean lifetime of the same BY-2 cells after NaCl stress. Lifetime StDev in range ±0.06-0.08 ns for BY-2:Control; ±0.1-0.13 ns for BY-2:Mann; ±0.1-0.16 ns for BY-2:NaCl**.**

.

|  | |  | |  | |  |
| --- | --- | --- | --- | --- | --- | --- |
|  | |  | |  | |  |
|  |  | |  | |  |  |
|  |  | |  | |  |  |
|  |  | |  | |  |  |
|  |  | |  | |  |  |
|  |  | |  | |  |  |
|  |  | |  | |  |  |
|  |  | |  | |  |  |
|  |  | |  | |  |  |
|  |  | |  | |  |  |
|  |  | |  | |  |  |
|  |  | |  | |  |  |
|  |  | |  | |  |  |
|  |  | |  | |  |  |
|  |  | |  | |  |  |
|  |  | |  | |  |  |
|  |  | |  | |  |  |
|  |  | |  | |  |  |
|  |  | |  | |  |  |
|  |  | |  | |  |  |
|  |  | |  | |  |  |
|  |  | |  | |  |  |
|  |  | |  | |  |  |
|  |  | |  | |  |  |
|  |  | |  | |  |  |
|  |  | |  | |  |  |
|  |  | |  | |  |  |
|  |  | |  | |  |  |
|  |  | |  | |  |  |
|  |  | |  | |  |  |
|  | |  | |  | |  |

.

| \|  \| \|  \|  \| \| --- \| --- \| --- \| --- \| \|  \|  \|  \|  \| \|  \|  \|  \|  \| \|  \|  \|  \|  \| \|  \|  \|  \|  \| \|  \|  \|  \|  \| \|  \|  \|  \|  \| \|  \|  \|  \|  \| \|  \|  \|  \|  \| \|  \|  \|  \|  \| \|  \|  \|  \|  \| \|  \|  \|  \|  \| \|  \|  \|  \|  \| \|  \|  \|  \|  \| \|  \|  \|  \|  \| |  |  |  |
| --- | --- | --- | --- | --- | --- | --- | --- | --- | --- | --- | --- | --- | --- | --- | --- | --- | --- | --- | --- | --- | --- | --- | --- | --- | --- | --- | --- | --- | --- | --- | --- | --- | --- | --- | --- | --- | --- | --- | --- | --- | --- | --- | --- | --- | --- | --- | --- | --- | --- | --- | --- | --- | --- | --- | --- | --- | --- | --- | --- | --- | --- | --- | --- |
|  |  |  |  |
