# Supplementary material for: The viscoelastic properties of Nicotiana tabacum BY-2 suspension cell lines adapted to high osmolarity
Source: BMC Plant Biol. 2025 Feb 25;25:255. doi: 10.1186/s12870-025-06232-3 (PMC11852555; doi:10.1186/s12870-025-06232-3)
Supplement: Supplementary file 2 — Supplementary Material 2 [file 12870_2025_6232_MOESM2_ESM.docx]

**Brillouin Spectroscopy for estimation of visco-elastic properties**

The BLS rely on scattering of light on spontaneously (thermally) activated density fluctuations which propagates throughout the material in form of the sound (pressure) waves called phonons [Dil, J.G. Brillouin scattering in condensed matter. Reports on Progress in Physics 45, 285-334 (1982); B. J. Berne and R. Pecora, Dynamic Light Scattering (J. Wiley & Sons, 1976).]. These waves acts as a source of uniaxial (longitudinal) stress which is completely internal in nature (generated inside sample without any external stimulus). The speed of acoustic waves, *c*_B_, depends on how the sample respond (how its volume changes) to the sound’s pressure (longitudinal stress), that is how compressible (or how rigid) it is

$c_{B}^{2}=1/\beta\rho=M'/\rho$ (S1)

where β is compressibility, M’ is the longitudinal rigidity modulus and ρ is mass density. Therefore, by measuring the speed of sound, the mechanical information (compressibility or rigidity) can be extracted.

The value of speed of sound is obtained indirectly and requires measurement of spectrum of light scattered on sound waves. Because sound wave is travelling with certain velocity, c_B_, the light being scattered has different frequency (because of the Doppler effect). As a result the spectrum shows characteristic signatures, so called Brillouin lines being spectrally shifted in relation to the frequency of incident light. The value of the Brillouin frequency shift, *f*_B_, can be extracted from the spectrum. This quantity is a measure of sound wave frequency. Translation of measured sound wave frequency, *f*_B_, to its velocity, *c*_B_, requires the acoustic wavelength, Λ

$c_{B}=f_{B}\Lambda$ (S2)

The wavelength Λ is set by particular geometry utilized during scattering experiment, which defines the scattering vector, q=2π/Λ. If scattered light is collected at an angle, θ, with respect to incident laser beam of wavelength λ and the scattering occurs in region characterized by refractive index *n*, then

$q=2\pi/\Lambda=\frac{4\pi n}{\lambda}\sin(\theta/2)$ (S3)

Combining eq.S2 with S3 the speed of sound is found

$c_{B}=\frac{f_{B}\lambda}{n\sin(\theta/2)}$ (S4)

which can then be used to estimate the value of compressibility, β, or modulus, M’, with eq.S1.

Combining eq.S1 and S4 shows that value of the Brillouin frequency shift, *f*_B_, is related to the longitudinal rigidity, refractive index and density

$f_{B}\sim\sqrt{\frac{n^{2}}{\rho}M'}$ (S5)

For full visco-elastic description of the material, in addition to sample rigidity (quantified by rigidity or storage modulus M’), also its viscosity is required (expressed in terms of the loss modulus M”). This information can be also extracted from Brillouin spectra. When acoustic wave travels through material, its energy is being gradually lost and the wave decays in time (and space). That is, the phonon has a finite lifetime. The material parameter controlling sound wave attenuation is the viscosity. The information on sample viscosity is encoded in the width of the Brillouin peak, g_B_.

$\eta_{B}=\frac{4\pi g_{B}}{q^{2}}$(S6)

**Brillouin spectra fitting**

The Brillouin spectrum is the dependence of the scattered light intensity, *I*, as a function of the difference between frequency of scattered and incident light, *f.* The Brillouin spectrum recorded from pure medium (Fig.1c) shows a single Brillouin line. To obtain the frequency shifts and linewidth, the spectra were fitted using hydrodynamic expression convoluted with the instrumental resolution function [Pecora].

$\begin{matrix} I(f)=A_{B}\left[\frac{g_{B}}{(f+f_{B})^{2}+g_{B}^{2}}+\frac{g_{B}}{(f-f_{B})^{2}+g_{B}^{2}}\right]+ \\ \frac{A_{B}g_{B}}{f_{B}}\left[\frac{f+f_{B}}{(f+f_{B})^{2}+g_{B}^{2}}-\frac{f-f_{B}}{(f-f_{B})^{2}+g_{B}^{2}}\right] \end{matrix}$ (S7)

In eq.S7 first two terms describe the Brillouin doublet composed of two lines symmetrically of amplitude A_B_ shifted at f_B_, with the width (half width at half maximum) g_B_, whereas the last two terms ensure the preservation of the first moment sum rule and only affect the symmetry of the Brillouin doublet.

When the Brillouin signal was acquired from within the cell (Fig.1d), we found that Eq.S7 does not provide a proper description. When observed in logarithmic scale, the spectrum is clearly composed of two peaks. The spectra were fitted with the two-phase model (a sum of two Brillouin doublets) convolved with the instrumental resolution function of TFPI.

(S8)

The meaning of the symbols in Eq.S8 is the same as for Eq.S7.

We found that the parameters (*f*_B_ and *g*_B_) of one of these peaks correspond to those found for pure medium. Therefore, we interpret the spectra as being recorded from a two-phase system. One phase corresponds to the medium surrounding cell and the other to the cell itself. The reason we see the signal from both phases (buffer and cell) is low optical resolution of our system given by a low numerical aperture of the objective together with a high diameter of entrance pinhole (200um).

The existence of a Brillouin signal from the cell and from the buffer was previously observed in microspectroscopic experiments utilising TFPI (Mattana et al., 2018). It is known to deliver spectra of exceptional quality and it is not clear if the same “buffer contamination” exists also in spectra recorded with VIPA spectrometers more commonly utilised by Brillouin imaging.

During fitting, the Brillouin line shape parameters for the buffer phase (*f*_B,buffer_ and *g*_B,buffer_) were kept constant and fixed to the value found from the fitting spectra acquired in pure media using eq.S7.

The parameters for the first Brillouin doublet were constrained to values obtained for the medium (*f*_B,buffer_ and *g*_B,buffer_). The fitting process involved free parameters, including the second phase parameters (*f*_B,cell_ and *g*_B,cell_) and the amplitude of the Brillouin peak of the medium (*A*_B,buffer_), following a similar approach presented in a prior study (Mattana et al., 2018).

**Estimation of cells elastic and vicious contrasts**

The values of both Brillouin line parameters (f_B_ and g_B_) measured inside the cells (belonging to different adaptation lines) are distinctly higher than those found in their respective pure media buffers (Fig.5). At the same time, these parameters measured for bulk media were also different.

To get meaningful information on the visco-elastic behaviour of the cells adapted to mechanically different environments, the Brillouin results were presented in the form of the relative (with respect to buffer) changes of Brillouin shift and Brillouin line width

$\begin{matrix} \upsilon_{B}=f_{B}/f_{\mathrm{B},buffer}-1 \\ \gamma_{B}=g_{\mathrm{B}}/g_{\mathrm{B},buffer}-1 \end{matrix}$ (S9)

We call such defined properties 'the elastic contrast' υ_B_, and “the viscous contrast”, γ_B_, (Bacete et al., 2021)).

It should be emphasised that the definitions of mechanical contrasts (elastic and viscous) adopted in this study differ from the usual ones (Antonacci et al., 2020), where changes in Brillouin line shape parameters are given in relation to the values characterising pure bulk water.

We decided to express the contrasts relative to the media, as these are the natural environments of the examined cells. Such defined mechanical contrasts can be interpreted in terms of change of internal cells composition being the result of cells adaptation to different environments.

The other directly measured parameter is the width of Brillouin line which informs about existence of some processes responsible for sound-wave attenuation. This parameter can be used to calculate the value of so called longitudinal viscosity (eq.S6). The biological origin of the longitudinal viscosity has not been systematically addressed. It was found, however, that changes in longitudinal viscosities can be related to changes in shear viscosity for aqueous solution of different biologically important solutes (Adichtchev et al., 2019). At a cellular level, the Brillouin linewidth may change as a result of liquid-to-solid phase transitions occurring in intracellular compartments (Antonacci et al., 2018), the presence of a lipid-rich layer surrounding amyloid plaques, as well as the level of hydration (Mattana et al., 2017) making longitudinal viscosity sensitive to molecular crowding. Correct interpretation of the BLS viscosity requires careful distinction of true viscous effects from other factors that lead to linewidth broadening, such as those caused by the use of a high numerical aperture objective (Antonacci et al., 2013) or potential existence of spatial mechanical inhomogeneities within the probed volume.
